# Performance improvement of haptic collision detection using subdivision surface and sphere clustering

**DOI:** 10.1371/journal.pone.0184334

**Published:** 2017-09-26

**Authors:** A. Ram Choi, Mee Young Sung

**Affiliations:** Department of Computer Science and Engineering, Incheon National University, Incheon, Korea; Universitat Rovira i Virgili, SPAIN

## Abstract

Haptics applications such as surgery simulations require collision detections that are more precise than others. An efficient collision detection method based on the clustering of bounding spheres was proposed in our prior study. This paper analyzes and compares the applied effects of the five most common subdivision surface methods on some 3D models for haptic collision detection. The five methods are Butterfly, Catmull-Clark, Mid-point, Loop, and LS3 (Least Squares Subdivision Surface). After performing a number of experiments, we have concluded that LS3 method is the most appropriate for haptic simulations. The more we applied surface subdivision, the more the collision detection results became precise. However, it is observed that the performance becomes better until a certain threshold and degrades afterward. In order to reduce the performance degradation, we adopted our prior work, which was the fast and precise collision detection method based on adaptive clustering. As a result, we obtained a notable improvement of the speed of collision detection.

## Introduction

Collision detection is a crucial issue that arises in haptics applications. If there is no collision detection, virtual objects would penetrate each other or will not be able to move them. Many algorithms have been proposed in previous decades to accelerate collision detection process. However, there are still some open challenges such as extremely high frequencies that are required for haptic rendering.

Haptic rendering is the core technology for haptic virtual reality system. By rendering, we refer to the process where the desired sensory stimuli are imposed on the user to convey information about a virtual haptic object [1]. The precise sensory feedback requires precise collision detection between a haptic device and a virtual haptic object. In addition, an ideal haptic rendering requires an update rate of 1000Hz.

In general, collision detection for interactive applications has to be done by filling in or covering target objects as tightly as possible with bounding volumes (spheres, axis-aligned bounding boxes, oriented bounding boxes, or polytopes). However, polygon level refined collision detection is also necessary for higher accuracy and stability. Because collision detection occurs on each polygon of the 3D model, it is reasonable that a 3D model with more refined polygons allows more accurate detectability.

In the field of 3D computer graphics, there is a modeling technique called “subdivision surfaces” [2] which are generating more refined smooth surfaces from a base polygonal mesh. It produces an approximation of a smooth surface by adding vertices and subdividing existing polygons through an iterative process that smoothens the mesh while increasing its density.

Subdivision surface schemes can be applied to any 3D model in an interactive haptic applications. Consequently, the study on the impact of subdivision surface for interactive haptic rendering is an interesting topic for improving the performance of collision detection.

This study is an extension of our prior work, “Controlling the Contact Levels of Detail for Fast and Precise Haptic Collision Detection” [3]. It proposes a method for creating bounding spheres with respect to the contact levels of detail (CLOD) which can fit objects while maintaining the balance between fast speed and precision of collision detection. A brief summary of this prior work is presented in one of the following sections.

The rest of this paper proceeds with a brief survey of related research including the properties of five typical methods for subdivision surfaces (Butterfly, Catmull-Clark, Loop, Mid-point, and LS3). Then, the analysis of the effects in graphical point of view (the appearance and the number of polygons) of the five subdivision surface methods, which are applied three times to a 3D graphics model, are presented. Next, the performance analysis in haptical point of view on the effects of five subdivision surface methods (the update rate, the collision detection speed, and the average triangle area) are elaborated. Then lastly, a number of improved results of the collision detection are presented, followed on by our discussion.

## Related works

In this section, we will briefly examine the basic concepts such as subdivision surface schemes, collision detection methods, and some other related works.

### Subdivision surfaces

Through the procedure of subdivision surfaces, we iteratively subdivide a rough surface or mesh into smaller faces to create a smooth surface, which also improves the approximation. As the process is repeated, new vertices and faces are formed within the original coarse mesh. The positions of the newly created vertices are chosen based on the positions of the other vertices in the vicinity.

Certain subdivision schemes allow change of the position of the original vertices to optimize the positions of the new ones. But commonly, as the result of subdivision surfaces, we increase the number of faces of the object, each with smaller area than the original mesh, creating a smoother and finer surface.

In a broad sense, there are two types of subdivision schemes: interpolation and approximation. Interpolating subdivisions require original vertices to be fixed in position as iteration goes on, whereas approximating subdivisions allow repositioning of the original vertices. In most cases, approximating schemes produce more smooth surfaces than interpolating subdivisions.

In general, subdivision surface refinement makes the surface everywhere smooth, however, many studies were conducted to represent piecewise smooth objects. Piecewise smooth objects consist of smoothly curved regions that meet along sharp curves and at sharp corners. At this present, many subdivision surface schemes have improved enough in order to represent sharp features using sharp subdivision rules or optimized multiresolution subdivision surfaces [4],[5],[6],[7].

#### Interpolating schemes

Interpolating subdivision surfaces make use of interpolation functions, e.g. Hermite spline function, to optimize the positions of new vertices in subdividing an original mesh into smaller faces. The scheme requires the original vertices or vertices from previous iterations (the control points) to be fixed in position when the optimal location of new vertices is being computed. Thus the final iteration (the limit surface) will have vertices that did not change their locations through the process. The benefit of this method is that it is relatively easy to reach the form of the final surface. One of the typical interpolating schemes is the Butterfly method [8].

#### Approximating schemes

Approximating subdivision surfaces uses B-spline function or Box-spline function to optimize the positions of newly created vertices. In this scheme, altering the positions of the original vertices or the vertices generated from previous iterations (the control points) are allowed, and the vertices located outside of the final surface (the limit surface) is shifted toward the limit surface. The benefit of this method is that the process creates less undulation or ripple phenomenon, and the end result is relatively smoother. But it is difficult to reach the final surface. Many methods including Catmull-Clark [9], Loop [10], Mid-point [11], and LS3 (Least Square Subdivision Surface) [12] belong to this scheme.

### Collision detection

Collision detection refers to the computational aspect of determining whether two objects have collided. Collision response refers to the simulation of the effect of a detected collision. So far, there are many important methods that have been developed for collision detection [13],[14],[15],[16],[17],[18],[19],[20],[21],[22],[23],[24],[25].

The study of Ruspini et al. [22] generates hierarchical bounding spheres using the balanced binary tree and multi-level approach. “High-level detection” of Ruspini’s work corresponds to the “fast detection” of the method proposed in our previous work, while “low-level detection” corresponds to the “precise detection” of ours. The experiments of Ruspini were conducted with the top-down algorithm, whereas ours were conducted with the bottom-up algorithm for sphere formation. The bottom-up approach can generate more precise and tighter bounding spheres because it starts from the terminal polygons.

The paper of Weller and Zachmann [23] is also written with a top-down approach, and their study uses a voxel-based algorithm and generates inner spheres, whereas ours generates outer spheres. The approach of wrapping with inner spheres often fail to envelop all of the boundary polygons, but our outer spheres approach can successfully do so.

Most of these methods can be directly applied to deformable objects. But the major drawback of the hierarchical collision detection algorithms is that the hierarchy has to be updated after every deformation. Consequently, much attention in the field has been devoted to developing hierarchies for easier computations.

#### Bounding volumes

To quickly detect collision between an object and a haptic pointer, a bounding volume of the object is necessary. In computational geometry and computer graphics, a bounding volume for a set of objects is a closed volume that can completely contain the union of objects in the set. Since simpler volumes normally have simpler ways to test for overlap, the method of using easier figures are used to improve the efficiency of geometrical operations.

To obtain bounding volumes of complex objects, a common way is to break the objects down using bounding volume hierarchies (BVHs). Bounding volumes for building hierarchies include bounding spheres (BSs) [13][14], axis-aligned bounding boxes (AABBs) [16], oriented bounding boxes (OBBs) [17], discrete orientation polytopes (k-DOPs) [18], etc. The basic idea behind these hierarchies is to organize a complex object in a tree-like structure where the root comprises the whole object and each leaf contains a smaller subpart. Collision detection is then carried out corresponding to the hierarchies of the bounding volumes.

Wrapping objects in bounding volumes and performing collision tests on them before testing the target geometry itself simplifies the tests and can significantly improve performance. By arranging the bounding volumes into a hierarchy, the time complexity (the number of tests performed) can be reduced to logarithms of the number of objects. With such structure in bounding volumes, if their parent volumes have not collided, children in the hierarchy do not have to be examined for collisions.

#### Bounding spheres

There are several fast and simple algorithms for constructing a bounding sphere volume with high practical value in real-time computer graphics applications. Since a sphere can be represented by its center and radius, it can be tested for collision with each other very quickly by simple checks on the radii. As two spheres intersect, the distance between their centers should not exceed the sum of their radii. This property makes bounding spheres applicable to objects in any dimension.

Bounding volumes are typically based on the space decomposition by tree data structures such as binary space partition (BSP) trees [26], k-d trees [27], octrees [28], R*-trees [29], grids [30], etc. Octrees are often used for partitioning a three-dimensional space into eight octants via recursive subdivision. However, a major limitation of such approach is that the leaf objects cannot usually be tightly enclosed because the number of leaf nodes is always fixed to eight. One of the methods that can resolve this problem is dividing the space using medial axis (topological skeleton).

The motivation for this approach is from Blum’s medial axis [31], which can be interpreted as a “skeleton” of a two-dimensional object. The medial axis or Voronoi skeleton of a polygon is the set of two or more neighboring points on the polygon’s boundary. A more technical definition involves the locus of points equidistant from two sides of the object. The three-dimensional version is called the medial axis surface. This structure contains surfaces rather than lines, but it remains analogous to a skeleton [15]. Our study for collision detection is based on this medial axis concept.

### Summary of our prior work

In our previous work, we proposed a method for creating bounding spheres with respect to the contact levels of detail (CLOD), which can fit objects while maintaining the balance between high speed and precision of collision detection. Our method is mainly composed of two parts: the bounding spheres formation, and two-level collision detection. The bounding sphere formation can be divided into two steps: creating spheres and clustering spheres. In addition, two-level collision detection is composed of fast detection of the sphere and precise detection in the sphere. As the first step of the process, bounding spheres are created for initial fast probing to detect collisions of spheres.

The motivation of our method can be found in the idea of distance-based clustering of bounding spheres for controlling the CLOD. The selection of the clustering distance depends on the update rate and the complexity of the base geometry. In addition, our algorithm reduces the average radii of spheres to enable a more precise detection of the object by implementing the centroid of a triangle, instead of using the circumcenter when calculating the center of a bounding sphere [3].

### Some comparative works

#### Haptic subdivision

Although there are many studies done in graphical subdivision surfaces, there are only a few studies conducted on haptic subdivision [32][33]. One of the most relevant works is a study which proposes a method for haptic subdivision which keeps the number of spheres under control so that the simulation can be run at a sufficiently high rate for force feedback [32]. Their work uses only the Loop subdivision surface method and calculates new values of mass and spring constants when a portion of the surface is subdivided. But ours analyzes five different methods for subdivision.

There is another study that concerns about the online re-mesh and multi-rate deformation simulation on a GPU (graphics processing unit) to concentrate the computational load into the regions that exhibit the most deformation [33]. This work suggests using a data structure which consists of an extended and transposed connection table, a sphere list, and a separated mass list. This study will be referenced for our future work which will deal with an efficient manipulation of CLOD in real-time.

#### Haptic smoothness

There are also some published works concerning haptic smoothness that can be compared with ours. The first one is, “Direct haptic rendering of sculptured models” [34] published in 1997 by T. V. Thompson II et al. This paper proposed direct haptic rendering composed of several phases such as surface proximity testing, tracking, contact and tracing, and transitions. This work concludes that their DPT (direct parametric tracing) method can improve the haptic rendering by exact computation of surface normals and higher order continuity of surfaces. However, this method only improves the calculation of haptic rendering instead of improving model meshes and does not consider any of the collision detection problems.

The second work is “Smooth force rendering on coarse polygonal meshes [35], published in 2010 by J. Wu et al. It locally constructs Gregory patches on the contact points and generates smooth haptic forces on coarse polygonal meshes without adding a heavy cost on computing time and memory usage. This method imposes some problem of discrepancy between the graphical sensation and the haptic sensation, because users may feel graphically coarse while feeling the object haptically smooth.

## Experiments for subdivision surfaces methods

Almost all 3D objects are represented as textured polygonal mesh models. It is because the polygonal graphical representation allows for fast and flexible rendering. However, polygons are planar and can hardly reflect the exact smoothness of the real surface. In order to approximate the real surface, the mesh surface has to be smoothened.

Subdivision surfaces are polygon mesh surfaces refined from a base mesh through an iterative process that smoothens the mesh while adding new vertices and faces. Therefore, complex smooth surfaces can be derived in a reasonably predictable way from relatively simple meshes.

**Fig 1:**
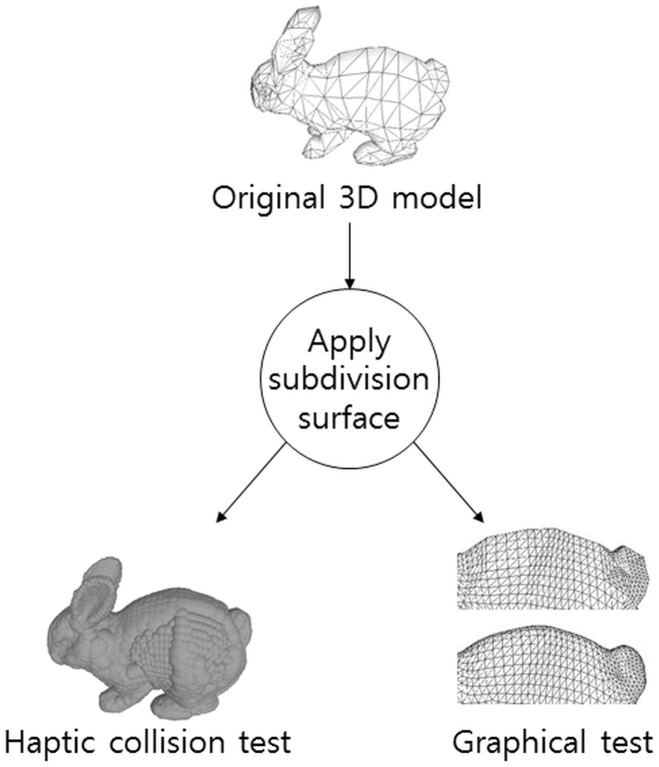
Comparison process flowchart.

We suppose that the mesh quality improvement can also affect the quality of our haptic collision detection method based on bounding sphere clustering. Subdivision surfaces will cause the change of directions of normal vectors and will influence the performance of the clustering of our collision detection method. Fig 1 illustrates the flow of comparison process.

Before describing our experiments, some terms are to be defined:

- *Precision* is the best numerical measure of its reliability which can be obtained after all known sources of error have been eliminated or corrected for [36].
- *Precision of graphical rendering* is the precision of the representation of each polygon of a 3D model.
- *Precision of haptic rendering* is the precision of the collision detection on each polygon of a 3D model.

In this study, the more precise haptic rendering means that the collision detection for haptic probing between an object and a haptic interface can be performed on a finer (smaller) sized polygon than the original polygon after some subdivision surface operations. In order to analyze different subdivision techniques, five most popular subdivision surface refinement methods, Butterfly, Catmull-Clark, Mid-point, Loop, and LS3 (Least Square Subdivision Surface), are examined.

For each category, we carried out the following two experiments for verifying the effects of subdivision surfaces:

1. Graphical test: Five subdivision surface methods are applied to a bunny mesh model [37] three times iteratively, in order to analyze the graphical characteristics of each subdivision surface method.
2. Haptic collision detection test: Five subdivision methods are examined for analyzing the computational complexity for collision detection. This test consists of the following criteria:
  - The graphical tightness of bounding spheres
  - The number of bounding spheres
  - The collision detection time
  - The update rate

It is mentionable that the first Graphical test measures, “How natural the model is”, which can be something subjective. The other four criteria of haptic collision test are measured quantitatively, which exhibit differences in respect to the original model and the average values in each of the 1st, 2nd, and 3rd steps of subdivision surfaces. Then the resulting values of differences are converted to the scores ranging from 0 to 5.

### Graphical test

First, we investigate the graphical effects of five different subdivision surface methods. The bunny mesh model was refined by applying the following subdivision surface methods:

1. Butterfly
2. Catmull-Clark
3. Mid-point
4. Loop
5. LS3

**Fig 2:**
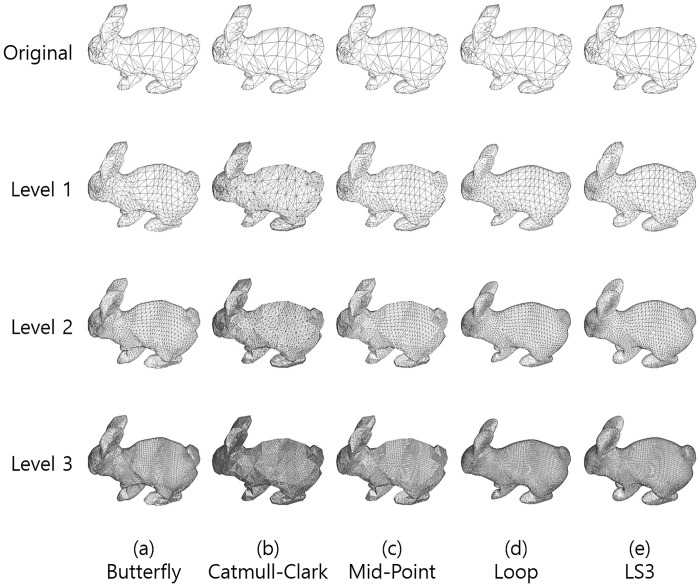
Results of subdivision surfaces by five different methods. From the left by each column, (a) Butterfly, (b) Catmull-Clark, (c) Mid-point, (d) Loop, (e) LS3 methods, from the top by each row, (1st row) the original meshes, (2nd row), (3rd row), and (4th row) correspond to the result of each level of subdivisions applied once, twice, and three times respectively.

The results are illustrated in Fig 2.

The Catmull-Clark method subdivides triangles while maintaining the original surfaces. Note that the shapes of triangles are generally elongated. The two sides of triangles are longer than the other and the number of vertices increases from 4.5 to 6 times. It is nearly double a amount of other methods.

The Mid-point method preserves the original surfaces as Catmull-Clark did, but the shapes of triangles are equilateral because the subdivisions take the exact mid-point of each two vertices. The number of vertices increases 3 or 4 times as Butterfly, Loop, LS3 methods did unlike the Catmull-Clark method.

The Butterfly, Loop, and LS3 methods refine surfaces smoother than the Catmull-Clark method and Mid-point method. The Butterfly method makes some dented regions. Loop method shrinks the model in general, and this shrinkage is noticeable in highly curved regions such as feet and ears, becoming more severe as the subdivision levels increase. The LS3 method significantly smoothens the model while preserving its shape. Therefore, LS3 is the best method among the five in terms of graphics.

In order to compare volumetric changes to the original volume of the bunny model with different subdivision methods and steps, we define a simple algorithm for approximating the volume of a model. This approximation corresponds to the simple summation of all tetrahedron volumes formed from the center of the model to all triangles of the model. The following outlines the algorithm for calculating the volume of a model.

/* Algorithm for approximating the volume of a model */

vector3 *center* = *object*.position

for each triangles *t*(i):

 vector3 *v0* = *t*(i).vertex0’s position

 vector3 *v1* = *t*(i).vertex1’s position

 vector3 *v2* = *t*(i).vertex2’s position

 vector3 *va* = *v1* –*v0*

 vector3 *vb*–*center*–*v0*

 vector3 *vc* = *v2* –*v0*

 *volume* + = |1 / 6 * (*va* × *vb* • *vc*)|

**Fig 3:**
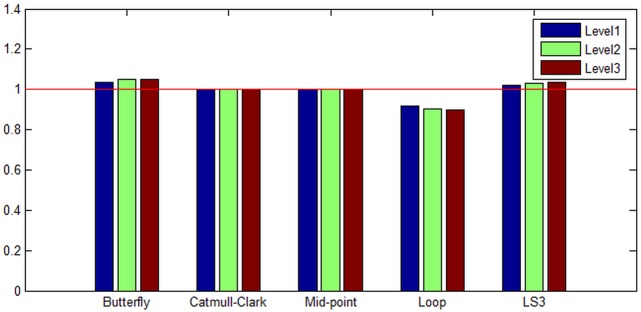
Bunny’s volume equality to original volume (set to 1). From the left, each column corresponds to Butterfly, Catmull-Clark, Mid-point, Loop, and LS3 methods with level 1, level 2, and level 3 of subdivision surfaces. Catmull-Clark and Mid-point are the best in volume equality while Loop is the worst in the category.

The results of the calculation of volumes for five methods in three levels of subdivision surface are presented in Fig 3. We call this experiment as the “volume equality” because the results signify how much equal to the original model whose volume is set to 1. According to the results, the Catmull-Clark and Mid-point give excellent volume equality (very little volume change), while the Loop method loses a great deal of volume.

### Collision detection test

In this section, we will discuss the effects of subdivision surfaces for our collision detection method which proposed in earlier work. Our collision detection method is based on the “clustering of bounding spheres” in respect to the CLOD [3].

Two categories of experiments are performed: one is *without clustering* and the other is *with clustering*. Concerning the clustering distance, we adopt 0.02 which is the optimal clustering distance for tooth, clover, and hat models in our experimental computing environment [3]. For each category, we analyze the following properties:

1. The graphical tightness of bounding spheres
2. The haptic collision detection measurements
  - The number of triangles
  - The average area of triangles
  - The number of bounding spheres
  - The collision detection time
  - The haptic update rate

#### Graphical tightness of bounding spheres

**Fig 4:**
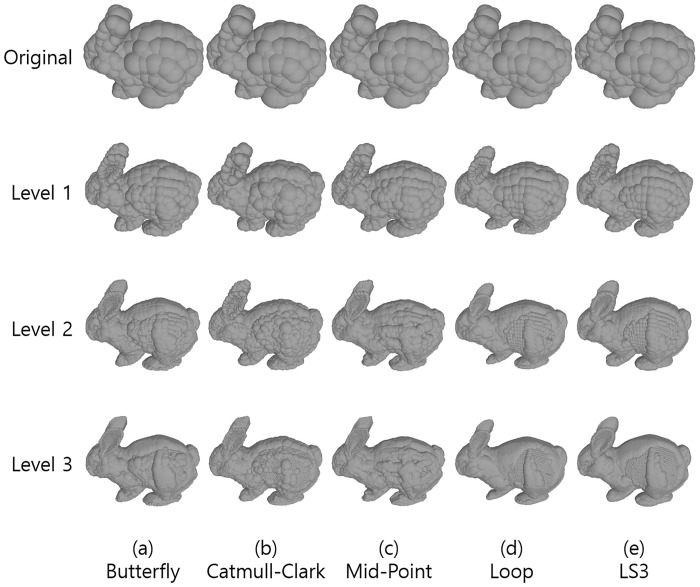
Bunny’s bounding spheres *without clustering*. From the left, each column corresponds to (a) Butterfly, (b) Catmull-Clark, (c) Mid-point, (d) Loop, (e) LS3 methods, from the top, each row corresponds to the original meshes, level 1, level 2, and level 3 of subdivision surfaces.

**Fig 5:**
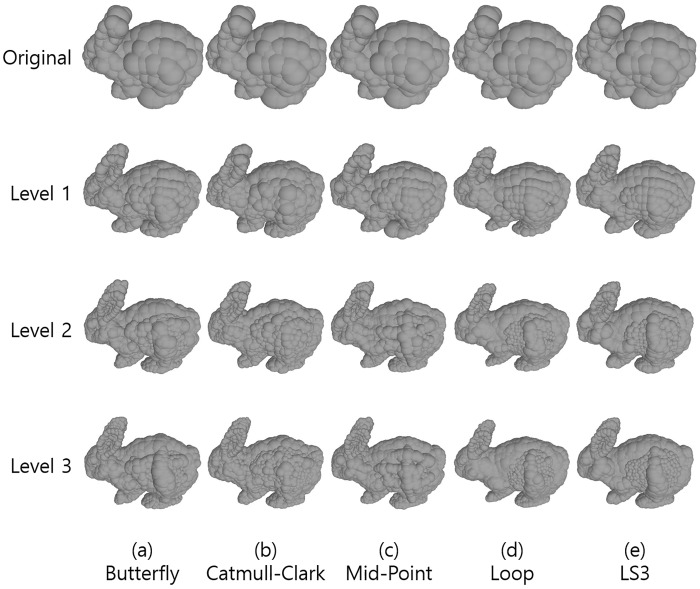
Bunny’s bounding spheres *with clustering (clustering distance is 0*.*02)*. From the left, each column corresponds to (a) Butterfly, (b) Catmull-Clark, (c) Mid-point, (d) Loop, (e) LS3 methods, from the top, each row corresponds to the original meshes, level 1, level 2, and level 3 of subdivision surfaces.

Five different subdivision surface methods are applied to Bunny model in three levels of subdivisions for analyzing the number of spheres for collision detection at each level of subdivision surfaces. Figs 4 and 5 illustrate the results of Bunny bounding spheres *without clustering* and *with clustering*, respectively. In Figs 4 and 5, from the left, the resulting bounding spheres are presented after applying (a) Butterfly, (b) Catmull-Clark, (c) Mid-point, (d) Loop, and (e) LS3 methods. From the top by each row, the 1^st^ row is the original meshes, and the 2^nd^ row corresponds to the 1^st^ level of subdivision surfaces, with the 3^rd^ row being the 2^nd^ level and so on.

The tightness of bounding spheres is a crucial criterion for collision detection. In order to compare tightness of bounding spheres to the original volume of the model, we define an algorithm which approximates the tightness of bounding spheres by calculating the difference between the centroids and the radius of bounding sphere as follows:

/* Algorithm to approximate tightness of bounding spheres */

for each bounding sphere*s*(i):

 for each triangle in the radius *t*(j):

  *tightness* + = *s*(i).radius–(*t*(j).centroid.distance(*s*(i).center))

  *num*++

*avgTightness* = *tightness* / *num*

**Fig 6:**
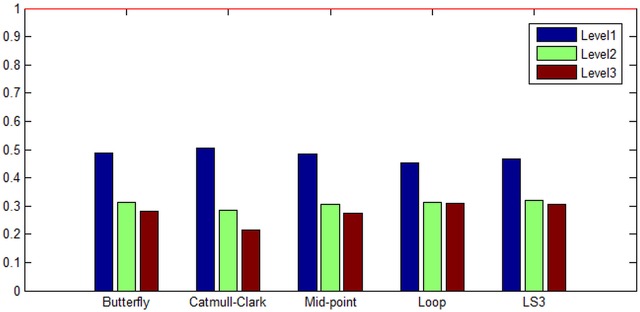
Bunny’s tightness of bounding spheres to original volume (set to 1). From the left, each column corresponds to Butterfly, Catmull-Clark, Mid-point, Loop, LS3 methods with level 1, level 2, and level 3 of subdivision surfaces. A value closer to 0 signifies better tightness. Accordingly, the Catmull-Clark method is the best in terms of tightness.

The value of average tightness which is closer to 0 signifies better tightness. It is evident that the volume of bounding spheres of a more refined model is tighter than the original one. However, the performance varies depending on different refinement methods. As shown in Fig 6, the Catmull-Clark method results in the best performance in level 2 and 3, and then Mid-point, Butterfly, LS3, Loop follow. Therefore, we can estimate that Catmull-Clark is a good method in the aspect of the graphical tightness of bounding spheres for collision detection.

#### Haptic collision detection measurements

Five subdivision surface methods are experimented for haptic probing on the Bunny model and gathered some collision detection measurements such as the number of triangles, the average area of triangles, the number of bounding spheres, the collision detection time, and the update rate.

Table 1 illustrates the results of Bunny’s haptic collision detection measurements *without clustering* and *with clustering*, after applying (a) Butterfly, (b) Catmull-Clark, (c) Mid-point, (d) Loop, and (e) LS3 in three levels of subdivision surfaces.

**Table 1:** Bunny’s haptic collision detection measurements. The number of average area of triangles, the number of spheres, the collision detection time in milliseconds, the update rate in Hertz *without clustering* and *with clustering* after applying (a) Butterfly, (b) Catmull-Clark, (c) Mid-point, (d) Loop, and (e) LS3 in three levels of subdivision surfaces. (The highlighted values are the values that show the best performance in each level.).

Bunny	Number of Triangles	Average Area of Triangles	Number of Bounding Spheres		Collision Detection Time (ms)		Update Rate (Hz)	
			without clustering	with clustering	without clustering	with clustering	without clustering	with clustering
Model	902	3.753	902	728	2.014	1.675	1095	1314
Butterfly(1)	**3580**	0.965	**3580**	1717	**2.452**	2.106	**581**	777
Butterfly(2)	13600	0.256	13600	2625	**3.149**	2.429	**416**	642
Butterfly(3)	41430	0.084	41430	3139	**4.183**	2.568	283	593
Catmull-Clark(1)	5412	**0.625**	5412	2128	2.890	2.295	501	692
Catmull-Clark(2)	21648	**0.156**	21648	3094	4.880	2.334	252	639
Catmull-Clark(3)	86592	**0.039**	86592	3827	10.453	2.954	102	505
Mid-point(1)	**3580**	0.946	**3580**	1704	2.778	1.997	521	834
Mid-point(2)	**13590**	0.249	**13590**	2551	3.300	**2.318**	399	669
Mid-point(3)	41120	0.082	41120	3049	4.408	2.551	275	621
Loop(1)	**3580**	0.852	**3580**	**1575**	2.574	**1.990**	576	**853**
Loop(2)	13600	0.220	13600	**2313**	3.774	2.319	327	**680**
Loop(3)	**38444**	0.077	**38444**	**2677**	6.757	**2.296**	**299**	**674**
LS3(1)	**3580**	0.939	**3580**	1686	2.620	2.140	562	797
LS3(2)	13668	0.247	13668	2469	3.466	2.517	374	642
LS3(3)	40730	0.083	40730	2896	8.019	2.619	139	601

**Fig 7:**
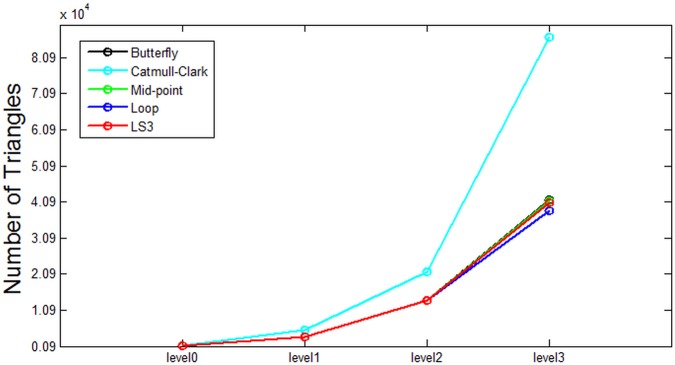
Comparison of the resulting number of triangles. Catmull-Clark method is the worst for the number of triangles in Bunny while the others show no significant difference.

Based on the data from Table 1, some analysis is presented in the following figures. Each figure from Fig 7 through Fig 11 corresponds to the analytical visualization of the number of triangles, the average area of triangles, the number of bounding spheres, the collision detection time, and the update rate.

**Fig 8:**
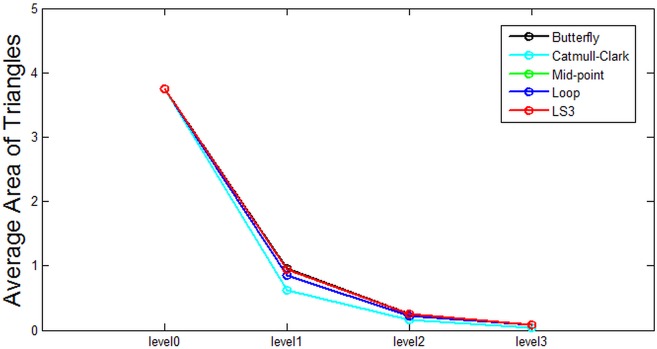
Comparison of the resulting average area of triangles. Catmull-Clark is the best performing method for the average area of a triangle on the Bunny.

**Fig 9:**
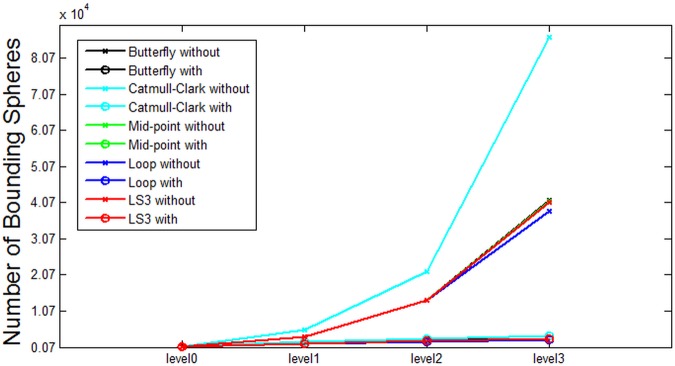
Comparison of the resulting number of bounding spheres *without clustering* and *with clustering*. Loop and LS3 show the best performance for Bunny’s number of bounding spheres.

**Fig 10:**
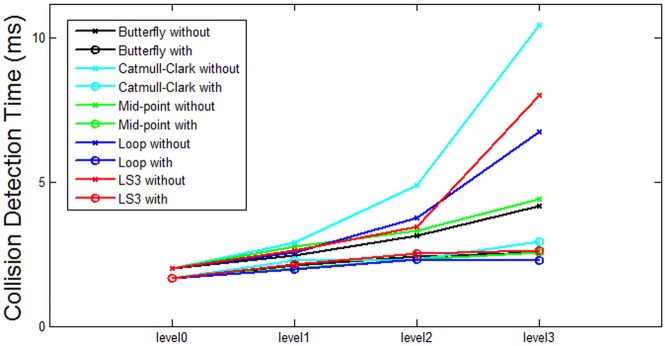
Comparison of the resulting collision detection time *without clustering* and *with clustering*. Loop is the best method for Bunny’s collision detection time.

**Fig 11:**
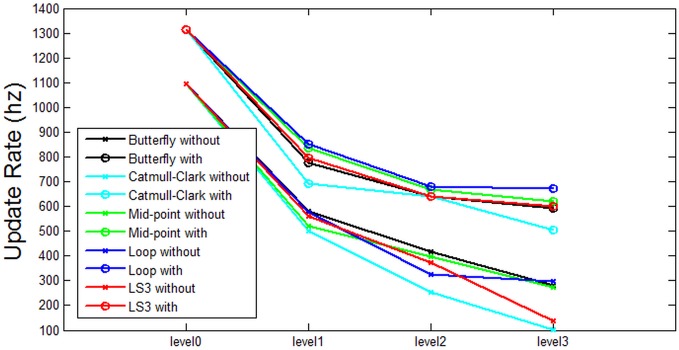
Comparison of the resulting update rates *without clustering* and *with clustering*. Loop with clustering is the best performing method for Bunny’s update rates.

The average area of triangles decreases as the subdivision continues. This means that the very precise collision detection is possible by examining the haptic contact to a very small triangular area.

As shown in Fig 9, the numbers of bounding spheres *without clustering* increase rapidly in proportion with the levels of subdivision, while the numbers of spheres *with clustering* increase slightly as the levels become higher.

Fig 10 summarizes the experiment on the collision detection time. It is remarkable that the growth rate of collision detection time without clustering is very steep, proportional to the levels of subdivision. On the contrary, the growth rate of collision detection time with clustering becomes more flat as the levels become higher.

In order to maintain a smooth and precise force feedback, the update rate for haptic rendering has to be more than 1000Hz. However, the update rates of all other cases except the original model are less than 1000Hz. Therefore, we may conclude that subdivision surfaces should not be applied if we want to keep the speed of haptic rendering. It means that some optimization for selecting the subdivision level is necessary for balancing the speed and the precision of collision detection if the subdivision surface is considered in haptic applications.

## Discussion

In this research, we performed a few experiments to analyze the effects of various surface subdivision on our collision detection. We analyzed the number of spheres, the collision detection time, average triangle area, and update rate for five general methods (Butterfly, Catmull-Clark, Mid-point, Loop, and LS3).

Firstly, we evaluated the five methods under numerous experimental criteria such as the graphical appearance, the volume equality, the tightness of bounding spheres, the collision detection time, and the update rates, with the scores ranging from 0 to 5. Table 2 shows the evaluation results where the best is the LS3 method. It can generate smoother surfaces as the steps of subdivision continues to be applied while keeping the original size of the model.

**Table 2:** Evaluation under various criteria. Graphical appearance, volume equality, tightness of bounding spheres, collision detection time, update rates.

Method	Graphical Appearance	Volume Equality	Tightness of Bounding Spheres	Collision Detection Time	Update Rate (Hz)	Total Score
Butterfly	2.00	4.77	3.20	3.65	2.74	16.36
Catmull-Clark	1.00	5.00	3.33	2.94	2.24	14.51
Mid-point	1.00	4.99	3.22	3.62	2.77	15.60
Loop	3.00	4.53	3.21	3.43	2.84	17.01
LS3	5.00	4.85	3.18	3.30	2.60	**18.93**

Secondly, we analyzed the effects of clustering of bounding spheres. In the cases of LS3, which is the best method for haptic subdivision surface, the clustering with the distance of 0.02 remarkably reduced the number of bounding spheres compared with the cases without clustering. For the example of the original bunny model summarized in Table 3, the number of spheres with clustering was reduced from 902 to 728 (reduction ratio of 19%). Then if the subdivision surface is applied once, it decreased from 3580 to 1686 (reduction ratio of 53%). If it is applied twice, it is decreased from 13668 to 2469 (reduction ratio of 82%). After applying the scheme thrice, it reduced from 40730 to 2896 (reduction ratio of 93%). Hence, the higher the level of subdivision, the higher the reduction ratio of the number of bounding spheres increases.

**Table 3:** Summary of LS3 cases.

Bunny	Number of Bounding Spheres			Collision Detection Time (ms)			Update Rate (Hz)		
	without clustering	with clustering	reduction ratio (%)	without clustering	with clustering	reduction ratio (%)	without clustering	with clustering	increment ratio (%)
Model	902	728	19	2.014	1.675	17	1095	1314	20
LS3(1)	3580	1686	53	2.620	2.140	18	562	797	42
LS3(2)	13668	2469	82	3.466	2.517	27	374	642	72
LS3(3)	40730	2896	93	8.019	2.619	67	139	601	332

In the matter of the ratio of the collision detection times with clustering, it reduced from 2.014ms to 1.675ms (reduction ratio of 17%) in the original model. If the LS3 subdivision is applied one time, it reduces from 2.620ms to 2.140ms (reduction ratio of 18%). When applied twice, it decreased from 3.466ms to 2.517ms (reduction ratio of 27%). When we applied it three times, it decreased from 8.019ms to 2.619ms (reduction ratio of 67%). Thus, the higher the level of subdivision, the reduction ratio of collision detection time also increases.

It is also important to examine the increment ratio of the update rate with clustering, which makes it rise from 1095Hz to 1314Hz (increment ratio of 20%) from the original model. If the subdivision is applied once, it changes from 562Hz to 797Hz, by 235Hz (increment ratio of 42%); when applied twice, it rises from 374Hz to 642Hz (increment ratio of 72%); when applied thrice, it increases from 139Hz to 601Hz (increment ratio of 332%). Similarly, the higher the level of subdivision, the higher the increment ratio of the update rate.

Table 3 summarizes the effect of clustering of bounding spheres by the application of LS3 subdivision surfaces to the Bunny model.

From the above analysis, we concluded that the performance of subdivision surface has trade-off relationships with that of collision detection algorithm. Subdivision surfaces can ensure precise collision detection. However, if the subdivision surface method is over-applied, the performance of collision detection degrades due to the calculation overhead. It might have occurred from the increased number of bounding spheres and their overlapping because of the exponential growth of the surface triangles, imposing unnecessarily many calculations. To alleviate this problem, we can apply the distance-based clustering technique, which is proposed in our prior studies. The experimental results summarized in Table 3 demonstrated that our distance-based clustering is able to improve the haptic rendering update rates and collision detection time, for example, 332% increment for update rates and 67% reduction for collision detection time in the case of the 3^rd^ level with LS3 method.

We also performed the same experiments for the tooth model and the clover model. In this paper, we only present the experimental results for the bunny model, because the results from the other models showed similar tendency. We intend to perform some similar experiments on other real-case models in the future.

In our subsequent works, we will focus on determining the optimal number of subdivision iterations which will maximize the collision detection speed while maintaining its precision. Note that all experiments are performed using the original version of each subdivision scheme. We will investigate and publish the result of the multiresolution haptic rendering for sharp features using improved subdivision surface schemes in future research.
